# Circular RNAs modulate cell death in cardiovascular diseases

**DOI:** 10.1038/s41420-025-02504-x

**Published:** 2025-05-02

**Authors:** Runfang Pan, Chinying Koo, Wenyuan Su, Qianhui You, Haidong Guo, Baonian Liu

**Affiliations:** 1 Department of Anatomy, School of Integrative Medicine, Shanghai University of Traditional Chinese Medicine, Shanghai, 201203 China; 2 Sport Medicine & Rehabilitation Center, Shanghai University of Sport, Shanghai, 200438 China

**Keywords:** Cell death, RNA splicing, Cardiovascular diseases

## Abstract

Cardiovascular diseases (CVDs) remain a global health challenge, with programmed cell death (PCD) mechanisms like apoptosis and necroptosis playing key roles in the progression. Circular RNAs (circRNAs) have recently been recognized as crucial regulators of gene expression, especially in modulating PCD. In current researches, circRNA regulation of apoptosis is the most studied area, followed by autophagy and ferroptosis. Notably, the regulatory role of circRNAs in pyroptosis and necroptosis has also begun to attract attention. From a mechanistic perspective, circRNAs influence cellular processes through several modes of action, including miRNA sponging, protein interactions, and polypeptide translation. Manipulating circRNAs and their downstream targets through inhibition or overexpression offers versatile therapeutic options for CVD treatment. Continued investigation into circRNA-mediated mechanisms may enhance our understanding of CVD pathophysiology and underscore their potential as novel and promising therapeutic targets.

## Facts

CircRNAs regulate multiple types of programmed cell death (PCD) in cardiovascular diseases (CVDs), including apoptosis, autophagy, ferroptosis, pyroptosis, and necroptosis, with apoptosis being the most extensively studied.

CircRNAs primarily function as molecular sponges for miRNAs, influencing signaling pathways such as NF-κB, PI3K/AKT, and TGF-β1, thereby affecting cardiomyocyte survival and death.

CircRNAs have potential as diagnostic and prognostic biomarkers in CVDs due to their stability, specificity, and detectability in extracellular vesicles.

Recent studies suggest that circRNAs play an essential role in regulating ferroptosis and its interaction with autophagy, offering a novel target for therapeutic intervention in conditions such as myocardial infarction and ischemia/reperfusion injury.

Despite advancements, challenges remain in understanding circRNA biogenesis, standardizing circRNA nomenclature, and translating circRNA-based research into clinical applications.

## Open Questions

What are the precise molecular mechanisms by which circRNAs regulate pyroptosis and necroptosis in CVDs, and how do these processes interact with other forms of PCD?

How can circRNA-based therapeutics be safely and effectively applied in clinical settings, minimizing off-target effects while maximizing therapeutic benefits?

How do circRNAs interact with other non-coding RNAs and protein networks to coordinate cell death regulation in CVDs?

## Introduction

Cardiovascular disease (CVD), which encompasses systemic vascular diseases affecting the heart and brain, poses a significant threat to human health [1] due to its high mortality, disability rates, recurrence rates, and numerous complications [2]. Despite advancements in research aimed at improving patient outcomes, clinical morbidity and mortality rates continue to rise [3, 4]. Consequently, there is a growing focus on discovering new treatment methods for CVD.

Programmed cell death (PCD), including apoptosis [5–13], necroptosis [14–16], pyroptosis [17–20], ferroptosis [6, 21–25], and autophagy-related cell death [26–29], is considered a key player in various cellular processes [30]. The abnormal activation of PCD pathways is implicated in the pathogenesis of various CVDs, such as ischemia/reperfusion (I/R) injury, myocardial infarction (MI), cardiomyopathy [31, 32] and atherosclerosis [33]. Accordingly, regulating cell death represents a significant potential measure for treating CVDs, and the timely activation of PCD can reshape the structure and function of the heart after injury [34].

Circular RNA (circRNA) is a large class of animal RNA with regulatory effects and typically shows tissue/developmental stage-specific expression [35]. Most circRNAs exhibit a high degree of conservation, while their expression patterns within individuals display tissue-specific or developmental specificity [36–38]. CircRNAs can regulate gene expression by serving as microRNA (miRNA) sponges or as scaffolds to facilitate contact between two or more proteins, thus representing potential regulators of cellular function [39–42]. Aberrant expression of circRNAs has been observed in certain CVDs like atherosclerosis, heart failure (HF), MI, and cardiomyopathy [43–48]. Studies also show that circRNA plays a key role in regulating cardiomyocyte apoptosis [49].

This article reviews the role of circRNA in CVD by regulating cell death patterns. It explores the origins, categorization, defining features, and functional roles of circRNA, and examines the distinctive attributes of PCD and its pivotal significance in cardiovascular ailments. We highlight the immense potential, future outlook, and therapeutic relevance of circRNA in modulating PCD to address CVD.

## Overview of CircRNA

CircRNA was first discovered in 1976 in plant viruses and demonstrated by electron microscopy in 1979 [50, 51]. CircRNAs consist of a large class of noncoding RNAs whose formation is associated with a back-splicing process that covalently links a downstream splice donor site to an upstream splice acceptor site [52]. Current research has established that circRNAs are derived from the cleavage of pre-mRNA by the spliceosome or group I and group II ribozymes, and the competitive dynamic between the formation of linear RNA and circRNA during transcription in eukaryotic cells is also proved [53]. However, the precise molecular mechanisms underlying circRNA biogenesis remain incompletely understood [54]. The lariat-or-exon skipping model and the direct backsplicing model are two major mechanisms in canonical spliceosomes [55]. Most circRNAs are derived from known protein-coding genes through back-splicing events, identified as exonic circRNAs (ecircRNAs), intronic RNAs (ciRNAs), and exon-intron circRNAs (EIciRNAs) [56]. EcircRNA functions in the cytoplasm, while ciRNA and EIciRNA primarily operate within the nucleus [57]. CiRNA enhances the transcription rate of target genes by regulating the elongation activity of the RNA polymerase II complex [58], and EIciRNA is a crucial factor in influencing differential gene expression between cells [58–60]. Mitochondria-derived circular RNAs (mecciRNAs) from animals were newly discovered in 2020, and there remains significant potential for further exploration. These ciRNAs can exert their functions in the mitochondria, nucleus, and cytoplasm [61–64]. Some studies have also investigated tRNA intron circRNAs (tricRNAs) [65], interior circRNAs (i-circRNAs) [66], antisense circRNAs [67–70], and intergenic circRNAs [56, 71, 72]. In summary, circRNAs primarily function as molecular sponges that sequester specific miRNAs, thereby targeting and regulating mRNA translation. Additionally, circRNAs act as protein sponges, to serve as auxiliary or regulatory molecules for proteins, mRNA, and DNA. They can also serve as templates for translation and scaffolds for nuclear translocation [73–75]. They exhibit remarkable stability against RNase R digestion, and are abundantly expressed in various organisms, and demonstrate specificity to tissues, diseases, and developmental stages. These characteristics endow circRNAs with significant potential as biomarkers.

## Circular RNAs modulate cell death in cardiovascular diseases

**Fig. 1:**
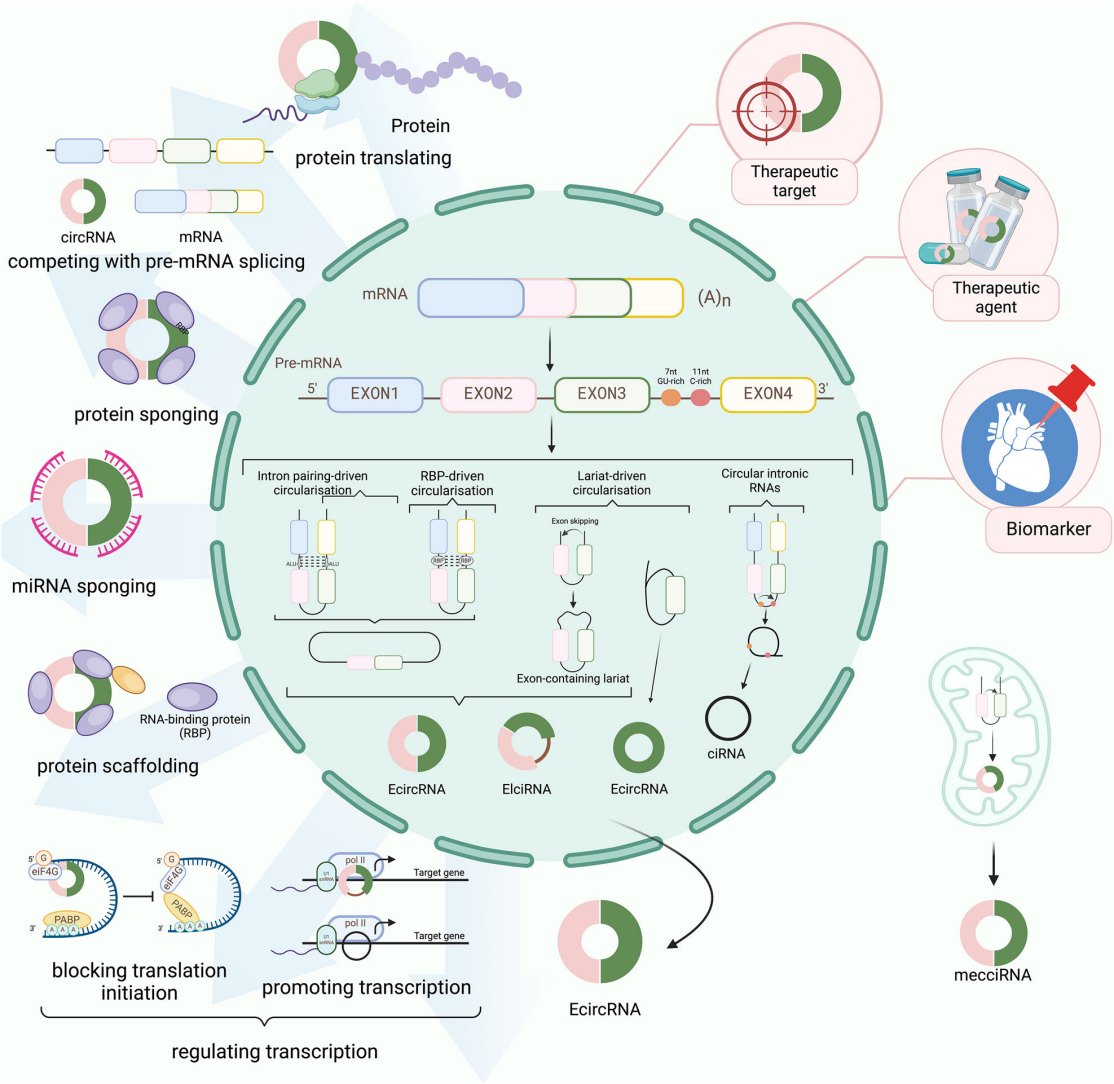
The biogenesis, subclasses, and functions of circRNAs (By Biorender). CircRNAs are non-coding RNAs formed via back-splicing, primarily through the lariat (exon-skipping) model or the direct back-splicing model. Based on their origin, circRNAs are categorized into EcircRNAs, ciRNAs, EIciRNAs, and mecciRNAs. They function through mechanisms such as miRNA sponging, regulation of pre-mRNA splicing, and protein scaffolding, with potential applications as therapeutic targets, therapeutic agents, and biomarkers.

CircRNAs, attributable to their stability and ability to circulate via exosomes, have shown promise as potential biomarkers for diagnosing and monitoring various diseases. There has been growing interest in its role in CVD in recent years. Studies on apoptosis are relatively abundant, demonstrating that circRNAs modulate apoptosis through miRNA sponging and signaling pathways such as TGF-β1 and NF-κB, providing flexible therapeutic strategies. Investigations into autophagy have revealed that it frequently coexists with apoptosis in target cells, with circRNAs playing key roles in modulating these processes, suggesting therapeutic potential for myocardial cells. In terms of ferroptosis, researchers highlight its impact on cardiomyocyte death and cardiac function, with a growing focus on circRNA-regulated signaling pathways, particularly in animal models. Though studies regarding pyroptosis are still limited, emerging findings suggest that circRNAs’ involvement in its regulation shows promise for clinical treatment. Similarly, while necroptosis research remains in its early stages, the regulatory role of circRNAs is becoming more apparent, offering potential new therapeutic targets for CVDs (Fig. 1).

### Apoptosis

Apoptosis is directly induced by the intrinsic BCL-2 pathway and the extrinsic death receptor pathway, where apoptotic factors promote the activation of the caspase cascade, leading to protein cleavage and eventual cell destruction [76]. Apoptosis continues to be a focus in various fields, including CVDs, cancer, neurological disorders, and liver diseases [77–82]. The role of circular RNAs (circRNAs) in regulating apoptosis in CVDs has attracted significant attention [83], with numerous studies conducted on various cardiovascular conditions such as atherosclerosis, AMI, and myocardial I/R injury.

In the context of atherosclerosis-related circRNAs, circRSF1, circ_0007478, hsa_circ_0004831, circ_0026218, circLZIC, circ_0065149, circ_0030042, circHIPK3, circRNA-0024103, circ_0010283, hsa_circ_0001445, circ_0005699, among others, generally reduce apoptosis and mitigate ox-LDL-induced vascular endothelial cell damage by sponging miRNAs and processing downstream signals [84–96]. The upregulation of circNRG-1 has been demonstrated to alleviate apoptosis in atherosclerosis and hypertension model cells treated with ANGII, while circANKRD42 and circUBAC2 are believed to mitigate apoptosis in the context of atherosclerosis and MI through multiple signaling pathways, including the circUBAC2/hsa-miR-200b-3p/HIPK3 axis and circANKRD42/hsa-miR-324-5p/AP1G1 axis [97, 98]. Circ_0003645, circ_0004104, circANRIL, circTEX14, circTM7SF3, circ_USP36 (hsa_circ_0003204), circ_0029589, circMTO1, circ_0124644, circ_0005699, circUSP36, circ_0021155, circ_0006476 primarily function through miRNA sponging, but their increased levels exacerbate apoptosis in atherosclerosis model cells [7, 11, 48, 99–108].

Several studies have focused on the role of circRNAs in regulating apoptosis in AMI, involving circSAMD4A, circ_0060745, circRBMS1, circUSP39, and circ_0008842. These circRNAs primarily exert their effects by sponging miRNAs, except circ_0060745, which acts through the NF-κB pathway. When these circRNAs are upregulated, they enhance apoptosis and worsen AMI [109–113].

Myocardial I/R injury is another research hotspot. CircRbms1 has been shown to increase apoptosis and exacerbate hypoxia-induced cardiomyocyte injury through the miR-2355-3p/MST1 and miR‑742‑3p/FOXO1 axis [114, 115]. Other circRNAs involved include circ_0050908, circARPA1, circRNA Fbxl5, circ-0001380, circ_SMG6, and circHIPK3, which regulate apoptosis levels through various signaling pathways, primarily by sponging miRNAs, thereby influencing myocardial I/R injury [13, 116–118].

Beyond the aforementioned conditions, studies on circRNA regulation of apoptosis in CVDs such as abdominal aortic aneurysm, aortic aneurysm, atrial fibrillation, cancer therapy-related cardiovascular toxicity, coronary artery disease, and MI are summarized in the table (Table 1) [75, 103, 114, 116, 117, 119–133].

**Table 1:** Circular RNAs modulate apoptosis in cardiovascular diseases.

Research model	Research subjects	Intervention methods	Circular RNA	Effects	Targets or pathways	References
Atherosclerosis	Human umbilical vein endothelial cells	In vitro: ox-LDL (150 µg/ml), 24 h	circ_0003645↓	Inflammation ↓ , apoptosis ↓ , cell viability rate ↓ , adhesion molecules↓	NF-κB ↑	M. Qin et al. [104]
Atherosclerosis	Human umbilical vein endothelial cells	In vitro: ox-LDL (100 μg/ml), 48 h	circRSF1↓	HUVEC growth ↓ , apoptosis ↑ , inflammation injury↑	miR-135b-5p ↑ /HDAC1↓ axis	X. Zhang et al. [84]
Atherosclerosis	Human Vascular smooth muscle cells	In vitro: ox-LDL (0, 25, 50, 100 µg/ml), 24 h	circ_0007478↓	Apoptosis ↑ , proliferation ↓ , migration ↓ , invasion↓	miR-638 ↓ , ROCK2↑	Z. Guan et al. [85]
Atherosclerosis	Human umbilical vein endothelial cells	In vitro: ox-LDL (100 mg/L), 24 h, ATV (10 μM), 24 h	hsa_circ_0004831↑	Apoptosis ↓ , oxidative stress ↓ , inflammation↓	miR-182-5p/CXCL12↑	G. Su et al. [86]
Atherosclerosis	Human umbilical vein endothelial cells	In vitro: ox-LDL (100 μg/ml), 24 h	circ_0004104↑	Dysfunction ↑ , cell viability ↓ , angiogenic ability ↓ , apoptosis ↑ , inflammation ↑ , oxidative stress↑	miR-328-3p ↓ /TRIM14↑ axis	C. Zhang et al. [99]
Atherosclerosis	SMC, CD68-positive macrophages	In vitro: ox-LDL	circANRIL↑	Exonuclease-mediated pre-rRNA ↑ , ribosome biogenesis ↑ , apoptosis ↑ , proliferation↓	Pescadillo Homoloue 1 (PES1)↓	L. Holdt et al. [48]
Atherosclerosis	Human aorta VSMCs	In vitro: ox-LDL	circTEX14↑	VSMCS ↓ , apoptosis↑	miR-6509-3p/THAP1↓	L. Kou et al. [7]
Atherosclerosis	Human acute monocytic leukemia cell, peripheral blood samples of AS patients	In vitro: ox-LDL (50 μg/ml), 24 h	circTM7SF3↑	Apoptosis ↑ , inflammation↑	miR-206 ↓ /ASPH axis↓	X. Wang et al. [11]
Atherosclerosis	Human umbilical vein endothelial cells	In vitro: ox-LDL (50 μg/ml), 24 h	circ_USP36 (hsa_circ_0003204)↑	Cell proliferation ability ↓ , apoptosis ↑ , inflammation↑	miR-197-3p/ROBO1 Axis ↓ , miR-98-5p/VCAM1 axis↑	Y. Zhang et al. [100]
Atherosclerosis	Vascular smooth muscle cells	In vitro: ox-LDL	circ_0029589↑	Proliferation ↓ , migration ↓ , invasion ↓ , apoptosis↑	miR-424-5p /IGF2 axis↓	H. Yu et al. [101]
Atherosclerosis	Vascular smooth muscle cells	In vitro: ox-LDL (50 μg/ml), 48 h	circMTO1 ↓	Apoptosis ↓ , proliferation ↑ , cell migration↑	miR-182-5p ↑ /RASA1↓ axis	N. Ji et al. [106]
Atherosclerosis	Human umbilical vein endothelial cells	In vitro: ox-LDL (100 μg/ml)	circ_0124644↑	Cyclind1 ↓ , apoptosis↑	miR-149-5p ↓ /PAPP-A↑ axis	G. Wang et al. [102]
Atherosclerosis	Human umbilical vein endothelial cells, Male C57BL/6 wild-type mice, ApoE-knockout mice	In vitro: ox-LDL (0, 25, 50 and 100 µg/ml), 0, 12, 24 and 48 h	circ_0005699↓	Apoptosis↓	miR-450b-5p ↑ /NFKB1↓signaling axis	T. Chen et al. [103]
Atherosclerosis	Human umbilical vein endothelial cells	In vitro: ox-LDL (0, 50, 75, and 100 μg/ml), 0, 24, 48, and 72 h	circ_0026218↑	Inflammatory response ↓ , oxidative stress ↓ , apoptosis↓	miR-338-3p ↓ , SIRT6↑	L. Yang et al. [88]
Atherosclerosis	Human umbilical vein endothelial cells, human AS clinical samples	In vitro: ox-LDL (100 μg/ml), 24 h	circLZIC↑	Cell activity ↑ , apoptosis↓	micro-330-5p ↓ /NOTCH2↑ signaling pathway	X. Men et al. [87]
Atherosclerosis	Human umbilical vein endothelial cells	In vitro: ox-LDL (60 µg/ml), 24 h	circUSP36↓	Cell cycle arrest ↓ , apoptosis↓	miR-20a-5p ↑ , ROCK2↓	J. Miao et al. [107]
Atherosclerosis	Human umbilical vein endothelial cells	In vitro: ox-LDL (100 μg/ml), 0, 3, 6, 12, 24, and 48 h	circ_0065149↑	Invasion capability ↑ , cell viability ↑ , apoptosis↓	miR-330-5p ↓ , NF-κBp65 ↓	D. Li et al. [94]
Atherosclerosis	Human umbilical vein endothelial cells	In vitro: ox-LDL (50 μg/ml), 24 h	circ_0030042↑	Cell proliferation ↑ , apoptosis ↓ , inflammation↓	miR-616-3p ↓ /RFX7↑ pathway	L. Yu et al. [93]
Atherosclerosis	VSMCS (HASMC, HUASMC)	In vitro: ox-LDL	circHIPK3↓	Cell proliferation ability ↓ , apoptosis↑	miR-637 ↑ /CDK6↓ axis	L. Kang et al. [92]
Atherosclerosis	Human umbilical vein endothelial cells, HEK293 T cells	In vitro: ox-LDL (0, 40, 60, 80, and 100 mg/l), 6 h, 12 h, 24 h, and 48 h	circRNA-0024103↑	Migration ↑ , invasion ↑ , tube-forming ability↑ apoptosis↓	miR-363 ↓ /MMP-10↑ axis	Y. Tian et al. [96]
Atherosclerosis	Human vascular smooth muscle cells	In vitro: ox-LDL (100 μg/ml)	circ_0010283↓	Proliferation ↓ , migration ↓ , invasion ↓ , apoptosis↑	miR-133a-3p ↑ /PAPPA↓ pathway	Z. Feng et al. [91]
Atherosclerosis	Fasting serum samples, elbow veins from patients with atherosclerosis, human aortic endothelial cells	In vitro: ox-LDL (25–200 μg/ml)	circRSF1↑	Cell viability ↑ , tube formation ↑ , migration ↑ , apoptosis↓	miR-758 ↓ /CCND2↑axis	Z. Wei et al. [90]
Atherosclerosis	Human umbilical vein endothelial cells	In vitro: ox-LDL (0, 10, 25, and 50 µg/ml), 0, 24, 48, and 72 h	hsa_circ_0001445↑	Inflammation ↓ , oxidative stress ↓ , apoptosis ↓ , proliferation↑	miRNA-640↓	Y. Cai et al. [89]
Atherosclerosis	Human aortic smooth muscle cells	In vitro: ox-LDL	circ_0021155↑	HASMC ↑ , SMMHC ↓ , VSMCs ↓ , apoptosis↑	miR-4459 ↓ , TRPM7 mRNA ↓ , miR-3689c↓	J. Lin et al. [108]
Atherosclerosis	Human THP-1 monocytes	In vitro: phorbol-12-myristate-13-acetate (100 ng/ml), 1 μM nicotine, 24 h	circ_0006476↑	Apoptosis↑	miR-3074-5p ↓ /DLL4 ↑ , the Notch signaling pathway↑	C. Lin et al. [105]
Atherosclerosis	Human umbilical vein endothelial cells	In vitro: ox-LDL (0, 25, 50 and 100 μg/mL), 24 h	circ_0005699↑	Apoptosis ↓ , viability ↑ , cell proliferation ↑ , angiogenesis ability ↑ , inflammatory response ↓ , oxidative stress↓	miR-384 ↓ /ASPH↑	X. Cao et al. [95]
Atherosclerosis and hypertension	Mesenteric artery smooth muscle cells, human embryonic kidney 293 A cells	In vitro: angiotensin II (ANGII, 10 − 7 M), 24 h	circNRG-1↓	Apoptosis ↑ , vascular remodeling↑	circNRG-1/miR-193b-5p/NRG-1↑ axis	Y. Sun et al. [97]
Atherosclerosis and myocardial infarction	Male C57BL/6NTac (WT) and ApoE^−/−^ mice, blood from patients with acute STEMI	In vivo: atherogenic high-fat diet (40% fat, 0.27% cholesterol)	circANKRD42 and circUBAC2↓	Apoptosis ↑ , infarct size↑	circUBAC2/hsa-miR-200b-3p/HIPK3 ↓ , circANKRD42/hsa-miR-324-5p/AP1G1 ↓ …	F. Holme et al. [98]
Abdominal aortic aneurysm	Human aortic smooth muscle cells	None	circCBFB↓	Apoptosis↑ proliferation↓	circCBFB ↓ /miR-28-5p ↑ /GRIA4 ↓ /LYPD3↓	J. Yue et al. [119]
Acute myocardial infarction	H9C2 cells, male C57BL/6 J mice	In vitro: hypoxia, 2 h In vivo: ligation, left anterior descending coronary artery (LAD)	circSAMD4A↑	Apoptosis ↑ , inflammatory response↑	miR-138-5p↓	X. Hu et al. [110]
Acute myocardial infarction	Male C57BL/6 J mice, 8–10 weeks	In vitro: hypoxia In vivo: ligation, LAD	circ_0060745↓	Myocardial infarct size ↓ , systolic cardiac functions ↑ , apoptosis ↓ , inflammation↓	NF-κB ↓	C. Zhai et al. [109]
Acute myocardial infarction	Male C57BL/6 mice, 8-week-old, H9c2 cells	In vitro: H_2_O_2_ In vivo: ligation, LAD	circRBMS1↓	Apoptosis ↓ , infarct size↓	miR-92a ↑ /BCL2L11↓ signaling pathway	L. Jin et al. [113]
Acute myocardial infarction	Human cardiomyocytes (AC16)	In vitro: hypoxia, 4 h	circUSP39↓	Malondialdehyde ↓ , apoptosis↓	miR-362-3p ↑ /TRAF3↓ axis	J. Wang et al. [112]
Acute myocardial infarction	Blood from patients with AMI, H9c2 cells	In vitro: hypoxia, 24 h	circ_0008842↑	Apoptosis ↓ , cell viability↑	miR-574-5p ↓ , CK-MB ↓ , cTnI↓	L. Zhang et al. [111]
Aortic aneurysm	Human aortic smooth muscle cells (V-SMC-6110), aortic tissues, patients with aortic aneurysm	None	hsa-circ-000595↓	Apoptosis ↓ , aortic aneurysm↑	miR-19a↑	C. Zheng et al. [120]
Atrial fibrillation	Human umbilical artery smooth muscle cells, human aortic smooth muscle cells	None	circFAT1(e2)↓	Cell viability ↓ , apoptosis↑	miR-298 ↑ /MYB↓ axis	Z. Shi et al. [121]
Cancer therapy-related cardiovascular toxicity	Male C57BL/6 J mice, 8-week-old	In vivo: Doxorubicin (5 mg/kg/wk), 4 weeks	circSorbs1↑	Apoptosis ↓ , myocardial proliferation↑	mir-99 ↓ /GATA4 ↑pathway	K. Huang et al. [133]
Coronary artery disease	Vascular smooth muscle cells	In vitro: Annexin V-FITC (5 μL), PI (5 μL)	circLDLR↑	Apoptosis ↑ , proliferation↓	miR-26-5p ↓ /KDM6A↑axis	H. Dai et al. [122]
Coronary artery disease	Human vascular endothelial cells	In vitro: ox-LDL (100 μg/ml), 24 h	circ_0004104↑	Proliferation ↓ , apoptosis ↑ , inflammation↑	miR-100/TNFAIP8 axis↓	N. Ji et al. [123]
Coronary artery disease	Blood, CAD patients (*N* = 23), Human cardiac microvascular endothelial cells	In vitro: ox-LDL (100 μg/ml), 24 h	circ_ROBO2↓	Apoptosis ↓ , cell angiogenic ability↑	miR-186-5p ↑ , Tripartite motif-containing 14 (TRIM14)↓	Q. Ye et al. [124]
Hypoxia-induced injury	Human cardiomyocytes (AC16)	In vitro: hypoxia	circHSPG2↓	Glycolysis ↑ , apoptosis ↓ , inflammation ↓ , oxidative stress↓	miR-1184 ↑ /MAP3K2↓ cascade	L. Huang et al. [9]
Ischemic heart disease	Primary cardiomyocytes, mouse cardiac fibroblasts, endothelial cell line YPEN, human MCF-7 cells, BALB/c mice (8 weeks)	In vivo: Doxorubicin (24 mg/kg in 8 injections over a period of 3 weeks via intraperitoneal administration)	circ-Amotl1↓	Cell proliferation ↑ , survival ↑ , apoptosis ↓ , LVEDD ↓ , LVESD ↓ , EF ↓ , FS ↓ , LVPW ↓ , LVP ↓ , HW/BW↓	pAKT↓	Y. Zeng et al. [75]
Ischemic insult	Adult ICR mice, 8 weeks, plasma samples, 30 healthy individuals and 30 patients with AMI	In vivo: ligation, LAD	circIGF1R↑	cardiomyocyte proliferation ↑ , apoptosis ↓ , cardiac dysfunction and fibrosis↓	circIGF1R/DDX5/β-catenin axis	T. Shan et al. [125]
Myocardial infarction	Male C57BL/6 mice, 8 weeks, mouse cardiac myocytes	In vitro: hypoxia, 24 h In vivo: ligation, LAD	Cdr1as (CIRS-7)↑	Apoptosis↑	miR-7a/b↓	H. Geng et al. [83]
Myocardial infarction	H9c2 Cells	In vitro: hypoxia	circMAT2B↓	Cell activity ↑ , apoptosis↓	miR-133 ↑ , PI3K/AKT ↑ , Raf/MEK/ERK↑	Y. Zhu et al. [126]
Myocardial infarction	Human cardiomyocytes (AC16)	In vitro: hypoxia, 96 h	circRNA MFACR↑	Apoptosis↑ myocardium injuries ↑ , infarct size↓	miR-125b↓	S. Wang et al. [127]
Myocardial infarction	Human cardiomyocytes (AC16)	In vitro: hypoxia, 24, 48, 72, and 96 h	circ ACAP2↑	Apoptosis↑	miR-532↑	J. Zhang et al. [128]
Myocardial infarction and hypoxia/reoxygenation injury	Male adult BALB/c mice	In vivo: ligation, LAD	circPVT1↓	Cell viability ↑ , proliferation ↑ , apoptosis ↓ , myocardial infarct size ↓ , fractional shortening ↑ , ejection fraction↑	P53 ↓ /TRAF6↑ pathway, SIRT7 ↓ , Keap1 ↓ /Nrf2↓pathway, PDCD4 ↓ , circPVT1/miR-125b/miR-200a axis↑	C. Luo et al. [129]
Myocardial infarction-induced myocardial fibrosis	Male Sprague-Dawley rats, aged 8 weeks old, weighing (200 ± 20) g	In vivo: ligation, left anterior descending coronary artery	circRNA 010567↑	Cardiac function ↑ , MF ↓ , LVEF ↑ , LVFS ↑ , LVDD ↓ , LVDS ↓ , apoptosis↓	TGF-β1 signaling pathway↓	M. Bai et al. [130]
Myocardial ischemia-reperfusion injury	Human-derived cardiomyocytes	In vitro: hypoxia, 6 h	circ_0050908↓	Apoptosis ↓ , inflammatory response ↓ , oxidative stress↓	miR-324-5p/TRAF3 axis↓	A. Jin et al. [117]
Myocardial ischemia-reperfusion injury	Primary human cardiac myocytes, male C57BL/6 mice	In vitro: hypoxia, 24 h	circRBMS1↓	Cardiac function damage ↓ , oxidative stress injury ↓ , inflammatory response ↓ , apoptosis↓	miR-2355-3p/ MST1 axis↓	Y. Liang et al. [114]
Myocardial ischemia-reperfusion injury	Mouse cardiomyocytes HL-1	In vitro: hypoxia, 6 h	circARPA1↑	Cardiomyocyte injury ↑ , cardiomyocyte fibrosis ↑ , apoptosis↑	miR-379-5p/KLF9 axis ↑ , Wnt/β-catenin signaling↑	X. Li et al. [116]
Myocardial ischemia-reperfusion injury	Male C57BL/6 mice	In vitro: hypoxia In vivo: surgery, ligated left anterior descending coronary artery	circRBMS1↑	Hypoxia-induced cardiomyocyte injury ↑ , apoptosis↑	miR-742-3p ↓ /FOXO1↑ axis	B. Liu et al. [115]
Myocardial ischemia-reperfusion injury	Male adult C57BL/6 mice, 8–10 weeks, neonatal mice ventricular myocytes	In vitro: hypoxia, 2 h In vivo: ischemia, 45 min	circRNA Fbxl5↓	Apoptosis ↓ , infarct size ↓ , cardiac function↑	miR-146a-MED1 axis↓	D. Li et al. [13]
Myocardial ischemia-reperfusion injury	Adult myocardial cell line HL-1	In vitro: hypoxia, 2 h	circ-0001380↓	Apoptosis ↓ , oxidative stress↓	miR-106b-5p/Phlpp2 axis↓	L. Wang et al. [118]
Myocardial ischemia-reperfusion injury	Male C57BL/6 mice	In vitro: hypoxia, 24 h In vivo: slipknot ligation, LAD	circ_SMG6↑	I/R injury ↑ , cardiac dysfunction ↑ , infarction area ↑ , pathological damage ↑ , apoptosis↑	miR-138-5p ↓ /EGR1 ↑ /TLR4/TRIF axis	H. Chen et al. [103]
Myocardial ischemia-reperfusion injury	Human-derived cardiomyocytes cells	In vitro: hypoxia, 10 h	circHIPK3↑	Apoptosis ↑ , proliferation↓	circHIPK3/miRNA-124-3p axis↓	M. Bai et al. [131]
Myocarditis	HCM cells	In vitro: LPS, 10 µg/ml, 48 h	circACSL1↑	Myocardial inflammation ↑ , myocardial injury ↑ , apoptosis↑	miR-8055↓	L. Zhang et al. [132]

**Fig. 2:**
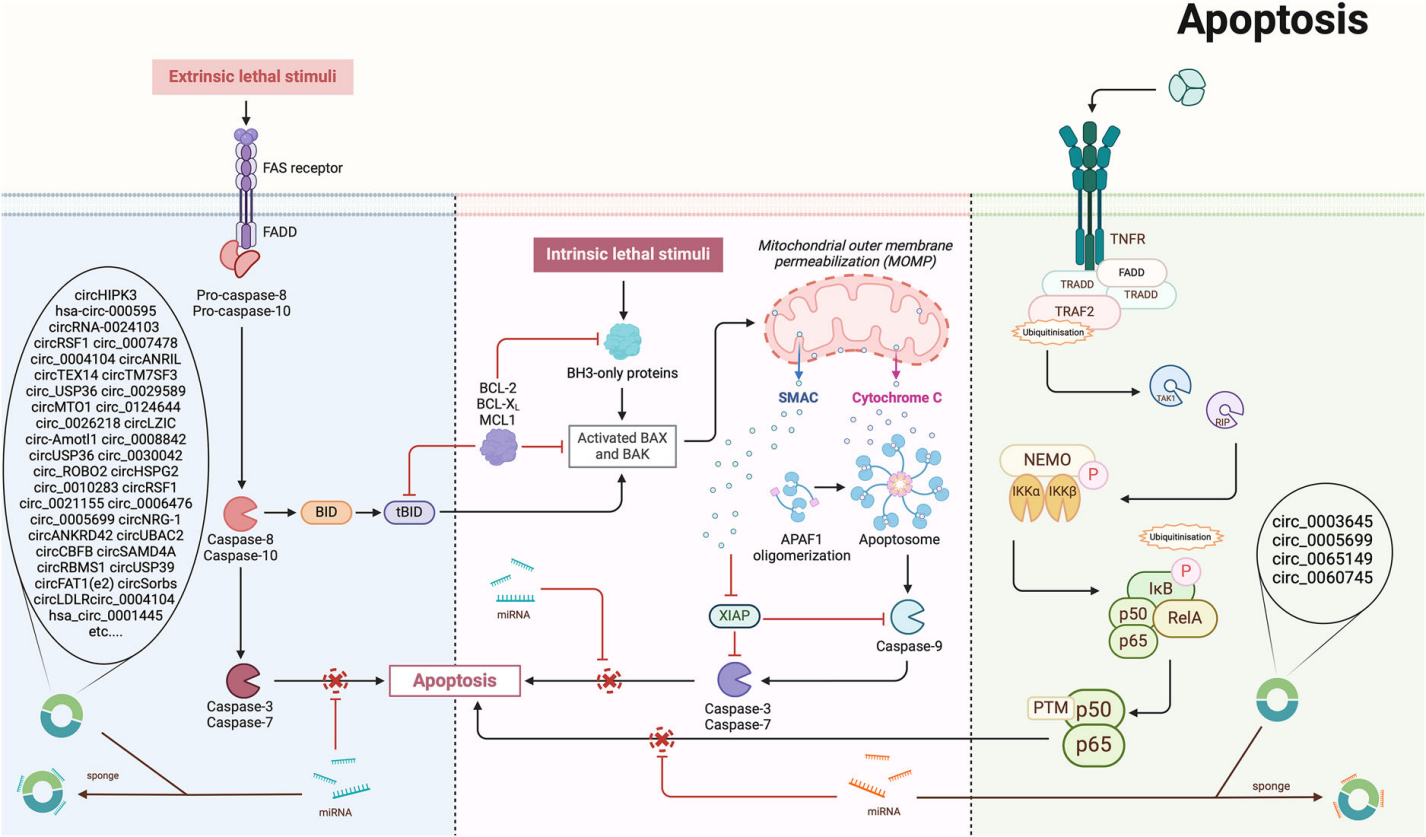
Mechanistic insights into circRNA-mediated regulation of apoptosis in cardiovascular diseases (By Biorender). CircRNAs influence apoptotic processes by interacting with specific miRNAs and modulating associated signaling cascades. Pro-apoptotic circRNAs intensify myocardial damage, while anti-apoptotic ones provide protective effects. The illustration highlights how various circRNAs contribute to the regulation of apoptosis during the progression of CVDs.

Currently, research on circRNA regulation of apoptosis in CVD treatment is relatively abundant. It is generally believed that circRNAs regulate apoptosis by sponging various corresponding miRNAs, as well as intervening in signaling pathways such as TGF-β1 and NF-κB. The inhibition or overexpression of specific circRNAs and their downstream molecular targets has different effects on apoptosis, offering flexible options in the clinical treatment of various CVDs (Fig. 2). This field has a solid research foundation and promising prospects for future studies.

### Autophagy

Autophagy refers to the process where autophagosomes encapsulate damaged proteins or organelles and transport them to lysosomes or vacuoles for degradation and recycling, typically to maintain homeostasis. Though, it is also implicated in cell death under certain circumstances [134]. Autophagy has garnered continuous attention in the fields of CVDs, cancer, neurodegenerative diseases, and metabolic disorders [135–140]. CircRNAs play a crucial role in regulating autophagy in CVDs [135, 141].

Studies have found that apoptosis and autophagy often occur simultaneously in the regulation of PCD in CVDs by circRNAs. CircRNA_101237 regulates IGF2BP3-dependent autophagy by sponging let-7a-5p, and the downregulation of IGF2BP3 resulting from circRNA_101237 downregulation reduces hypoxia/reoxygenation (H/R)-induced cardiomyocyte apoptosis and inhibits autophagy [140]. Ox-LDL-induced atherosclerosis remains a popular research focus. Circ_0002331 can enhance CCND2 activity to reduce autophagy and apoptosis, while circSQSTM1 exerts its effects through two pathways: one involves sponging miR-23b-3p, leading to increased Sirt1 expression, and the other enhances Sirt1 via the eIF4A3/FOXO1/Sirt1 axis [142, 143]. Knockdown of circPAN3 effectively alleviates autophagy and apoptosis, and improves cardiac function in MI mice via the miR-221/PTEN/AKT/PI3K pathway [144]. Circ-HIPK2 positively regulates ATG101 expression by sponging miR-485-5p, to accelerate apoptosis and cell death in myocardial oxidative damage induced by H_2_O_2_ [29]. Silencing circ_0010729 increases the viability of primary mouse cardiomyocytes and reduces OGD-induced myocardial cell injury by inhibiting apoptosis and autophagy through the miR-338-3p/CALM2 axis [145].

Research has proposed that circ-SIRT1 can promote autophagy in human-induced pluripotent stem cell-derived cardiomyocytes (hiPSC-CMs) and H9c2 cardiomyocytes by activating SIRT1, thus mitigating Ang II-induced cardiac hypertrophy (CH), based on the finding that autophagy deficiency leads to CH [28].

In patients with coronary heart disease (CHD), hsa_circ_0030042 is significantly downregulated in peripheral blood. In ApoE^−/−^ mice fed a high-fat diet, hsa_circ_0030042 counteracts eIF4A3-induced plaque instability and inhibits autophagy by sponging eIF4A3, preventing it from recruiting beclin1 and FOXO1 mRNA [27].

CircRNA ACR inhibits autophagy via the ACR-Pink1-FAM65B axis, where Pink1 suppresses autophagy and its downstream target FAM65B. When phosphorylated by Pink1, it also inhibits autophagy and cell death in the heart. ACR reduces myocardial I/R injury and MI area through this signaling pathway [146].

**Fig. 3:**
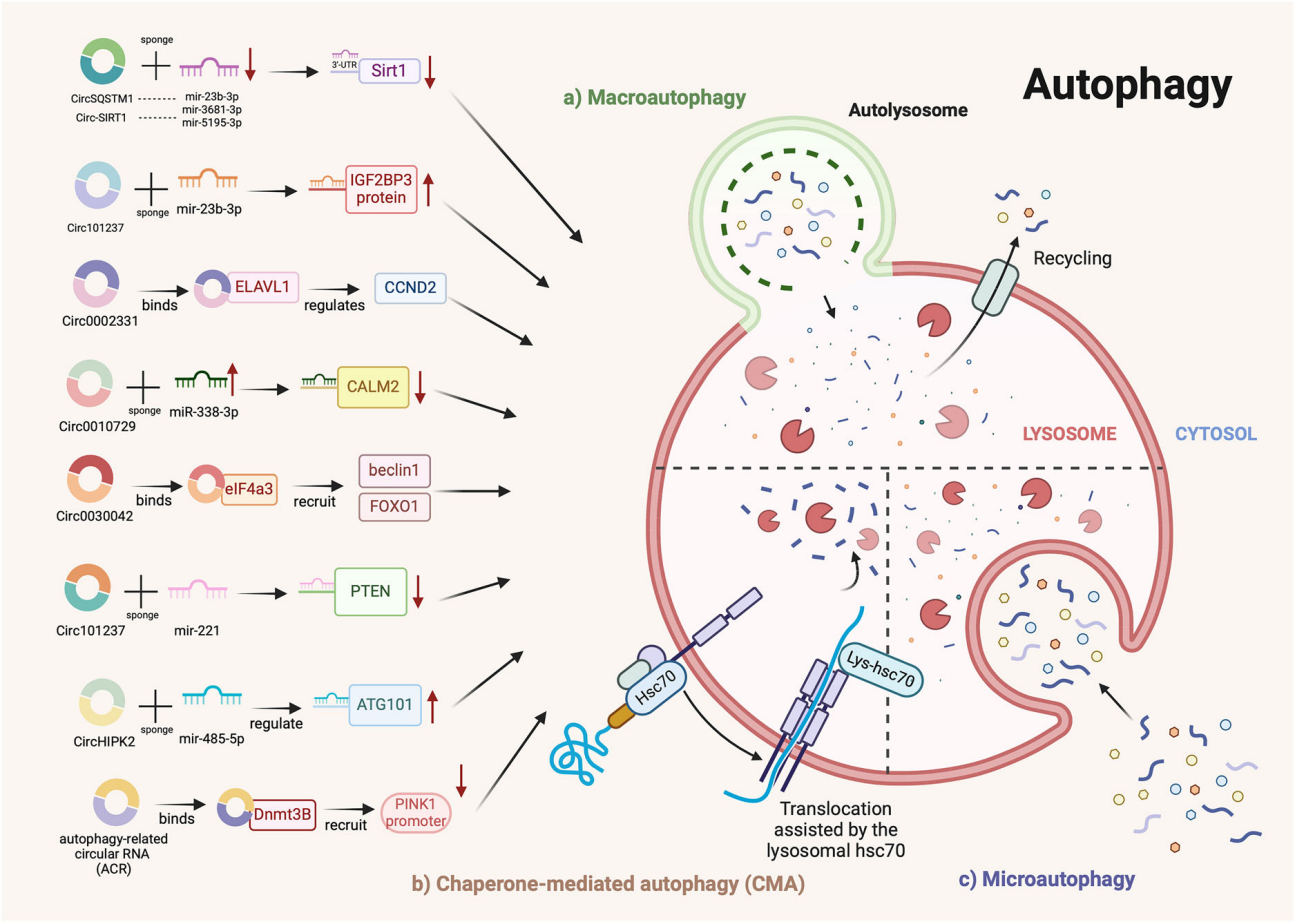
Mechanistic pathways of circRNA involvement in autophagy regulation in cardiovascular diseases (By Biorender). CircRNAs modulate autophagic activity by acting as miRNA sponges and altering the activity of critical signaling pathways. The diagram depicts the roles of circRNAs in regulating three distinct forms of autophagy: (a) macroautophagy, (b) chaperone-mediated autophagy, and (c) microautophagy, within the context of CVDs.

Several studies on circRNA regulation of CVDs through autophagy have noted that apoptosis and autophagy frequently coexist in target cells [147–149]. Furthermore, the exploration of circRNA regulatory mechanisms on autophagy and their downstream signals has advanced, particularly in deciphering molecular pathways associated with their miRNA-sponging functions (Fig. 3) [150–153]. The promotion or inhibition of autophagy by circRNAs may hold potential therapeutic value for myocardial cells in various CVDs.

### Ferroptosis

Ferroptosis is a form of iron-dependent PCD in which the accumulation of lipid peroxides and reactive oxygen species generated by iron metabolism leads to lipid membrane damage and cell failure [154]. Ferroptosis continues to attract attention in the fields of CVDs, tumors, inflammation, kidney damage, etc. [155–160]. Circular RNA regulates ferroptosis and plays an important role in CVD [161, 162].

The link between autophagy and ferroptosis is gaining attention in the study of the molecular mechanisms by which circRNAs regulate CVDs. CircRNA1615 regulates the expression of LRP6 by sponging miR-152-3p, thereby preventing LRP6-mediated autophagy-associated ferroptosis in cardiomyocytes through the miRNA152-3p/LRP6 molecular axis, ultimately controlling the pathological process of MI [24].

CircPIK3C2A promotes ferroptosis in AIC-treated H9c2 cells by sponging miR-31-5p, which upregulates TFRC, thereby exacerbating I/R injury [163]. Circ_0091761 enhances ferroptosis and reduces cell viability in H9c2 cells under simulated heart failure conditions through the miR-335-3p/ASCL4 axis and the TFRC axis [164]. Similarly, circ_005077 aggravates the adverse cardiac effects in various myocardial lipotoxicity models by enhancing ferroptosis, but its effects are not mediated by sponging miRNA. Instead, it upregulates CyPA and downregulates p47PHOX [165].

Overexpression of circRNA FEACR can suppress H/R-induced ferroptosis, inhibit MI, and improve cardiac function. This effect is mediated through the circRNA FEACR-induced NAMPT-Sirt1-FOXO1-FTH1 signaling axis. FEACR and its downstream factors can be considered novel targets for mitigating ferroptosis-related MI in ischemic heart disease [25].

In a pathological CH model using transverse aortic constriction (TAC) mice, circCmss1 was significantly increased in normal TAC mice but decreased in NSD2^−/−^ TAC mice. CircCmss1 interacts with the transcription factor EIF4A3 to induce the expression of transferrin receptor 1 (TfR1), thereby activating ferroptosis in cardiomyocytes [166].

The potential circRNA target for heart failure, circSnx12, can act as an endogenous sponge binding to miR-224-5p and regulating the miRNA binding site in the 3’UTR region of FTH1. Knockdown of circSnx12 increases ferroptosis, which is associated with mitochondrial abnormalities and myocardial cell death, exacerbating heart failure [167].

**Fig. 4:**
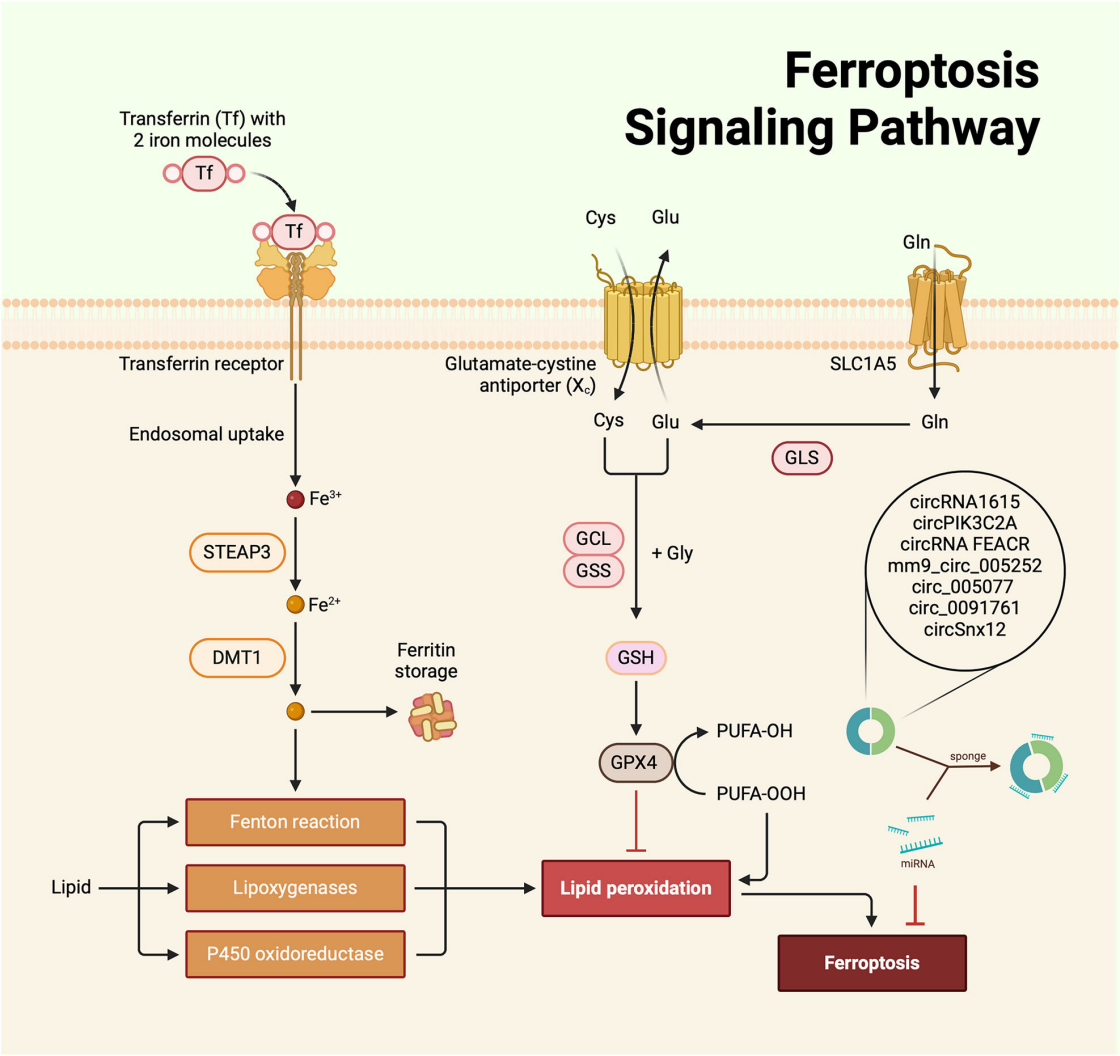
Role of circRNAs in ferroptosis regulation during cardiovascular disease progression (By Biorender). Through their interaction with specific miRNAs and modulation of key signaling networks, circRNAs exert regulatory control over ferroptosis. The figure illustrates the involvement of various circRNAs in the regulation of ferroptotic pathways in CVDs.

The role of circRNA-regulated ferroptosis in CVDs focuses on affecting cardiac function through the induction of cell death in cardiomyocytes and other cells. Current research frequently employs animal models of corresponding CVDs. The combined regulation of autophagy and ferroptosis by circRNAs is also receiving significant attention. Molecular information in this process, such as signaling axes involved in circRNA-regulated ferroptosis in CVDs, is gradually being elucidated, offering substantial targeted therapeutic potential for CVDs (Fig. 4).

### Pyroptosis

Cell pyroptosis is a form of PCD associated with inflammatory responses [168]. Pyroptosis can be triggered by oxidative stress, hyperglycemia, inflammation, and other stimuli [169]. It is dependent on caspase-1 and is characterized by the release of large amounts of pro-inflammatory factors, making it a form of apoptosis specific to single cells [170]. Pyroptosis continues to receive widespread attention in the fields of CVDs, cancer, and metabolic diseases [171–174]. Its role in disease mechanisms, particularly through causing inflammation and tissue damage, is especially emphasized. Pyroptosis plays a crucial role in CVDs [175]. Currently, research on circRNA regulation of pyroptosis in the field of CVDs is relatively limited, primarily focusing on atherosclerosis, diabetic cardiomyopathy, heart failure, I/R injury, and MI.

In atherosclerosis mouse models and HUVECs treated with ox-LDL, upregulation of circ-USP9× has been observed. Circ-USP9× promotes ox-LDL-induced pyroptosis in HUVECs by binding to EIF4A3 and enhancing GSDMD stability in the cytoplasm. Conversely, the knockdown of circ-USP9× inhibits ox-LDL-induced pyroptosis in HUVECs [176]. CircRNA DICAR is considered an important endogenous regulator of diabetic cardiomyopathy and cardiomyocyte pyroptosis, functioning through DICAR-VCP-Med12 degradation. Studies have found that DICAR-deficient (DICAR^+/−^) mice exhibit spontaneous cardiac dysfunction and abnormal cardiomyocyte morphology, and DICAR knockout also enhances diabetic cardiomyocyte pyroptosis. Clinical samples have shown that DICAR expression in the circulating blood of diabetic patients is lower than in healthy controls [46].

The sponging function of circRNAs is significant in regulating pyroptosis. In the context of heart failure, circ-0006332 has been found to exacerbate pyroptosis and apoptosis, while also worsening cardiac dysfunction and myocardial fibrosis. This effect is mediated by sponging miR-143, leading to the upregulation of TLR2 [177]. As mentioned earlier, knockdown of circPAN3 has been shown to effectively alleviate autophagy and apoptosis, while another study focused on the reduction of pyroptosis following circPAN3 knockdown under I/R injury conditions, achieved by decreasing its sponging of miR-29b-3p [178]. CircDGKZ, via the miR-345-5p/TLR4/NF-κB axis, reduces pyroptosis while increasing autophagy, thereby mitigating I/R injury [179]. In cardiomyocytes, miR-133a-3p inhibit NLRP3 inflammasome activation induced by MI, while circHelz acts as an endogenous sponge for miR-133a-3p, inhibiting its activity and enhancing pyroptosis. CircHelz can also directly trigger NLRP3 inflammasome-mediated pro-inflammatory responses, causing MI. Therefore, silencing circHelz holds potential therapeutic value for alleviating ischemic heart disease [180].

**Fig. 5:**
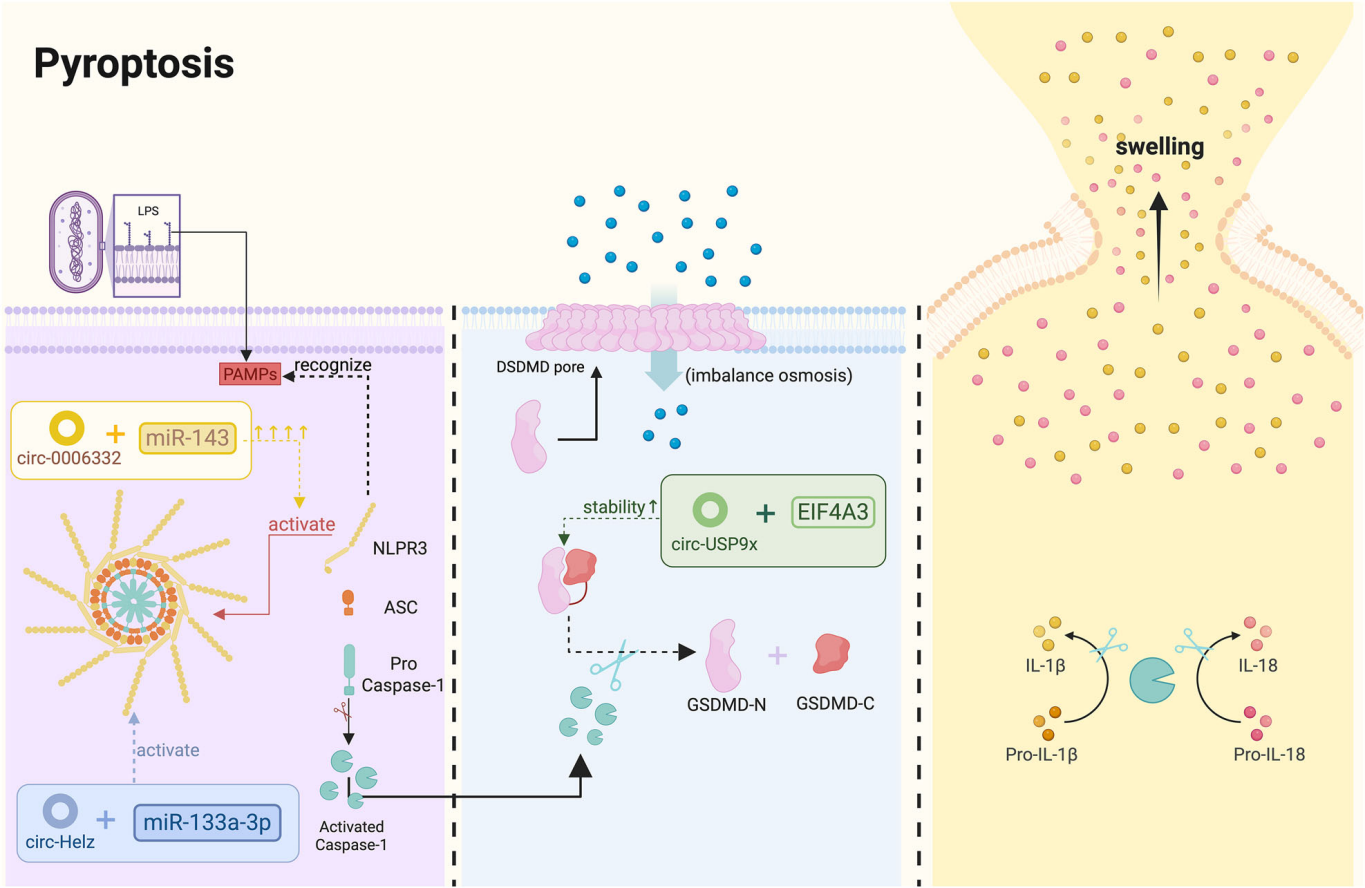
Mechanistic regulation of pyroptosis by circRNAs in cardiovascular diseases (By Biorender). Cell pyroptosis is dependent on caspase-1 and is characterized by the release of large amounts of pro-inflammatory factors, with or without sponging miRNA. The figure highlights pro-pyroptotic circRNAs in the context of CVD progression like circ-0006332, circ-Helz and circ-USP9x, underscoring their therapeutic potential.

Although the number of studies on circRNA regulation of pyroptosis in CVD treatment is still limited, the potential regulatory mechanisms of circRNAs on pyroptosis have gradually been uncovered. CircRNAs may serve as candidate drugs for the clinical treatment of CVDs or as upstream molecular signals, demonstrating significant research potential (Fig. 5).

### Necroptosis

Necroptosis is a pathway independent of caspase activation, characterized morphologically by distinctive plasma membrane rupture, and induces an inflammatory response [181, 182]. Necrosis continues to attract attention in the fields of CVDs, tumors, neurological disorders, renal injury, and other diseases [155, 160, 183–187]. At present, there is a relatively limited amount of research on circRNA regulation of necroptosis in the field of CVDs.

In H_2_O_2_-induced necroptosis of human aorta smooth muscle cells, an upregulation of circHIPK3 was observed. CircHIPK3 impairs mitochondrial energy production and induces cell death by acting on DRP1, but its mechanism of action on mitochondrial function is independent of DRP1 abundance. In vivo experiments revealed that the downregulation of circHIPK3 can regulate necroptosis and vulnerable plaque formation in ApoE^−/−^ mice, making it a promising therapeutic target for atherosclerosis [188].

Mmu_circ_000338, a cardiac-necroptosis-associated circRNA, is observed to be downregulated in cardiomyocytes exposed to H/R and in the hearts of mice with I/R injury. Overexpression of CNEACR reduces necroptosis and improves cardiac function in I/R injured hearts. The CNEACR/HDAC7/Foxa2/RIPK3 axis involved in this process may serve as an effective target for mitigating MI caused by necroptosis in ischemic heart disease (Table 2) [189].

**Table 2:** Circular RNAs modulate autophagy, ferroptosis, pyroptosis, and necroptosis in cardiovascular diseases.

Research Model	Research Subjects	Intervention Methods	Cell death type	Circular RNA	Effects	Targets or pathways	References
Anoxia/reoxygenation	Cardiomyocytes, 2-day-old mice	In vitro: hypoxia, 24 h	Autophagy and apoptosis	circRNA_101237↓	Autophagy ↓ , apoptosis↓	Let-7a-5p/IGF2BP3 axis↓	J. Gan et al. [140]
Atherosclerosis	Human umbilical vein endothelial cells, ApoE^−/−^ male mice aged 8 weeks	In vitro: ox-LDL (50 μg/mL), 24 h, ATV (0.1 μmol/L) and fluvastatin (1 μmol/L) and pitavastatin (1 μmol/L) and pravastatin (10 μmol/L) and simvastatin (1 μmol/L), 24 h	Autophagy	circSQSTM1↑	Autophagy ↑ , inflammation ↓ , oxidative stress↓	miR-23b-3p ↓ / Sirt1 ↑ , eIF4A3/ FOXO1/Sirt1 ↑ , YTHDF2↓	Z. Chen et al. [142]
Atherosclerosis	Human umbilical vein endothelial cells	In vitro: ox-LDL (100 mg/L), 24 h	Autophagy and apoptosis	circ_0002331↑	Autophagy ↓ , apoptosis ↓ , inflammation↓	CCND2↑	F. Chen et al. [143]
Cardiac hypertrophy	Human-induced pluripotent stem cell-derived cardiomyocytes, H9c2 cardiomyocytes, male C57BL/6 mice	In vitro: Ang II (150 nm), 24 h Isoproterenol (50 μM), 24 h In vivo: Ang II (N = 6)	Autophagy	circ-SIRT1 (hsa_circ_0093884)↑	Autophagy ↑ , SIRT1 ↑ , CH formation↓	miR-3681-3p ↓ , miR-5195-3p ↓ , USP22↑	W. Wang et al. [28]
Cardiac myocytes injury	Human cardiomyocytes (AC16)	In vitro: OGD, 6 h	Autophagy and apoptosis	circ_0010729↓	Cell viability ↑ , apoptosis ↓ , autophagy↓	miR-338-3p ↑ /CALM2↓axis	B. Ma et al. [145]
Coronary heart disease	Human coronary cells, ApoE^−/−^ male mice, 8-week-old	In vivo: atherogenic diet In vitro: ox-LDL (100 µg/ml)	Autophagy	hsa_circ_0030042↑	Autophagy ↓ , plaque stability ↑ , autophagosomes ↑ , PS exposure↑	4A-III (eIF4a3)↓, beclin1 ↓ , FOXO1↓	F. Yu et al. (2021) [226]
Myocardial infarction	Wistar male rats, 6-8 weeks old	In vivo: LAD ligation, quercetin (30 mg/kg) peritoneal injection, every other day, 2 weeks	Autophagy and apoptosis	circPAN3↓	Atuophagy ↓ , apoptosis ↓ , cardiac function↑	circPAN3 ↓ /miR-221 ↑ /PTEN↓ pathway, PTEN ↓ /AKT ↑ /PI3K↑ pathway	M. Farazi et al. [144]
Myocardial injury	Primary mouse neonatal cardiomyocytes, C57BL/6 mice of 1–3 days	In vitro: H_2_O_2_ (100, 200, 300, or 400 μmol/L), 12 h	Autophagy and apoptosis	circ-HIPK2↑	Cell proliferation ↓ , apoptosis ↑ , autophagy ↑ , IF intensity↑	miR-485-5p ↓ /ATG101↑ pathway	J. Zhou et al. [29]
Myocardial ischemia-reperfusion injury	Cardiomyocytes, 1 to 2 days old mice	In vitro: anoxia	Autophagy	circRNA ACR↑	Autophagy ↓ , cell death ↓ , extensive vacuolization↓	ACR-Pink1-FAM65B axis↑	L. Zhou [146]
Myocardial infarction	C57BL/6J male mice, 8–10 weeks old, mouse cardiomyocytes (HL-1 cells)	In vitro: erastin (0, 2.5, 5, 10, 20, and 40 μM), ZVAD-FMK (10 μM), necrosulfonamide (0.5 μM) In vivo: Ferroptosis inhibitor Fer-1 (1 mg/kg)	Ferroptosis and autophagy	circRNA1615↓	Ferroptosis↑	miRNA152-3p ↑ /LRP6↓ molecular axis	R. Li et al. [24]
Myocardial injury	H9c2 cells	In vitro: AIC (500 μM), 24 h	Ferroptosis	circPIK3C2A↑	Ferroptosis ↑ , I/R injury↑	circPIK3C2A ↑ /miR-31-5p ↓ /TFRC↑ axis	S. Miao et al. [163]
Myocardial ischemia-reperfusion injury	Primary cardiomyocytes, newborn mice aged 1–3 days, C57BL/6 J mice (8–10 weeks old)	In vitro: hypoxia, 18 h In vivo: ligation, left anterior descending coronary artery (LAD), 45 min, Adv-FEACR, adv-NAMPT, and adv-FTH1 (2 × 10^10^ moi)	Ferroptosis	circRNA FEACR↑	Ferroptosis ↓ , I/R-induced myocardial infarction↓	NAMPT-Sirt1-FOXO1-FTH1↑	J. Ju et al. [25]
Myocardial lipotoxicity	male Wistar rats	In vitro: PA (150 μM), 48 h In vivo: HFD (60% ratio of fat to energy supply)	Ferroptosis	circ_005077↑	Ferroptosis ↑ , hypertrophy ↑ , cardiac dysfunction ↑ , myocardial hypertrophy ↑ , fibrosis↑	CyPA ↑ /p47PHOX↓	X. Ni et al. [165]
Pathological myocardial hypertrophy	NSD2^fl/fl^-α-MHC^Cre+^ transgenic mice	In vitro: Ang II (200 nmol/l), 48 h In vivo: ligation, aorta	Ferroptosis	mm9_circ_005252 (circCmss1)↓	Cardiac hypertrophy ↓ , ferroptosis↑	circCmss1/EIF4A3/TfR1↓	Q. Xu et al. [166]
Heart Failure	Male C57/BL6J mice, six-week-old	In vivo: ligation, aorta	Ferroptosis	circSnx12↓	ferroptosis ↑ , mitochondrial abnormalities ↑ , myocardial cell death↑	miR-224-5p ↑ , FTH1↓	H. Zheng et al. [167]
Heart failure	H9c2 cells	In vitro: hypoxia, 3 h	Ferroptosis	circ_0091761↑	ferroptosis ↑ , cell viability↓	microRNA-335-3p ↓ / ASCL4↑ axis, TFRC axis↑	W. Qian et al. [164]
Atherosclerosis	Apo E^−/−^ mice, human umbilical vein endothelial cells	In vitro: ox-LDL (100 μg/ml), 12 h In vivo: High-fat diet (HFD), 15 weeks	Pyroptosis	circ-USP9×↓	Pyroptosis ↓ , cell death↓	EIF4A3 ↑ , GSDMD↓	S. Xu et al. [166]
Diabetic cardiomyopathy	DICAR^+/−^ mice and DICAR^Tg^ mice	None	Pyroptosis	circRNA DICAR↓	Pyroptosis↑	VCP ↓ , MED12↓	Q. Yuan et al. [46]
Heart failure	H9C2 cells, male Sprague-Dawley rats (aged 8–10 weeks)	In vitro: DOX (1 μg/mL), 24 h In vivo: intraperitoneal injection, DOX (2.5 mg/kg), once a week, 6 weeks	Pyroptosis and apoptosis	circ-0006332↑	Pyroptosis ↑ , cardiac dysfunction ↑ , myocardial apoptosis ↑ , myocardial fibrosis↑	miR-143 ↓ /TLR2↑ axis	P. Zhang et al. [177]
Ischemia/reperfusion injury	male Sprague Dawley rats (220–250 g), human cardiomyocytes	In vitro: hypoxia, 4 h In vivo: ligation, left coronary artery	Pyroptosis and apoptosis	circPAN3↓	Pyroptosis ↓ , apoptosis ↓ , myocardial infarct size ↓ , left ventricular end-diastolic pressure↓	circPAN3 ↓ /miR-29b-3p ↑ /SDF4↓ axis	A. Li et al. [46]
Ischemia/reperfusion injury	Eight-week-old wild-type (WT) male SD rats, human AC16 cardiomyocytes	In vitro: hypoxia In vivo: ligation, pulmonary arteries	Pyroptosis and autophagy	circDGKZ↓	Pyroptosis ↓ , autophagy ↑ , myocardial ischaemia–reperfusion↓	miR-345-5p ↑ /TLR4 ↓ / NF-κB ↓	S. Li et al. [179]
Myocardial infarction	Male C57BL/6 mice, 8 weeks old	In vivo: ligation, left anterior descending artery (LAD)	Pyroptosis	circHelz↑	Pyroptosis↑	miR-133a-3p ↓ / NLRP3↑	Y. Bian et al. [180]
Atherosclerosis	ApoE^−/−^ male mice (C57BL/6 J background, 6-weeks-old), human aorta smooth muscle cells	In vitro: 500 μM H_2_O_2_, 24 h In vivo: high fat diet, 6 weeks	Necroptosis	circHIPK3↑	mitochondrial division rate ↑ , reactive oxygen species ↑ , mitochondrial function ↓ , necroptosis↑	circHIPK3/DRP1	X. Li et al. [188]
Myocardial ischemia-reperfusion injury	I/R-injured mice hearts	None	Necroptosis	mmu_circ_000338 (CNEACR)↑	Myocardial infarction size ↑ , cardiac function ↑ , necroptosis↓	CNEACR/HDAC7 ↓ /Foxa2 ↑ / RIPK3↓ axis	X. Gao et al. [189]

**Fig. 6:**
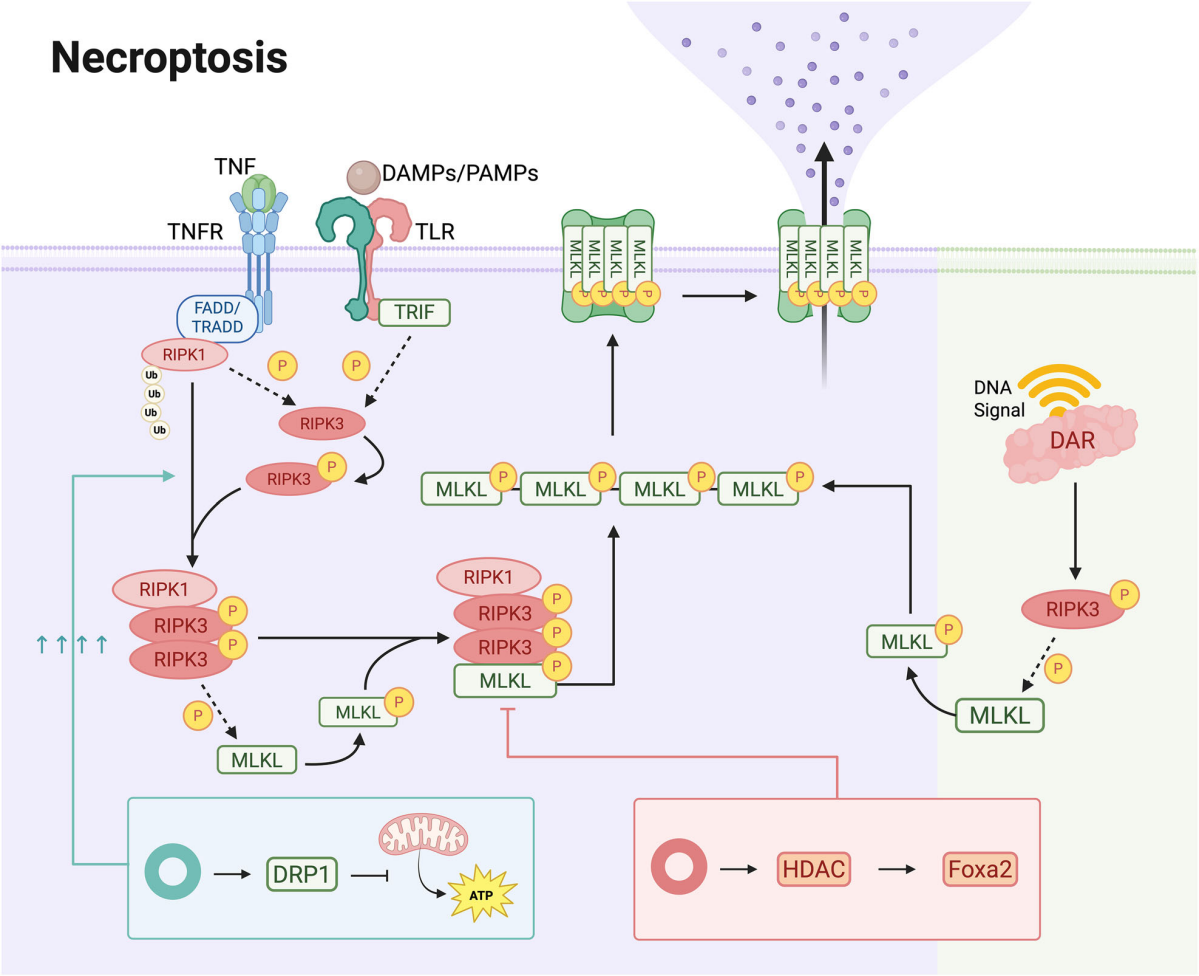
CircRNA-mediated regulation of necroptosis in cardiovascular diseases (By Biorender). Necroptosis is a caspase-independent cell death pathway associated with membrane rupture and inflammation. The figure depicts circRNA-regulated necroptotic signaling, including DRP1-mediated mitochondrial dysfunction and the Foxa2/RIPK3 axis, emphasizing circRNAs as emerging modulators in CVD pathogenesis.

The research on the regulation of necroptosis by circRNAs in CVDs is still in its early stages, involving relatively few CVDs and circRNAs. However, necroptosis has garnered extensive research attention across various disease fields, including CVDs. The signaling pathways by which circRNAs regulate necroptosis are gradually being elucidated, and the application of circRNA regulation of necroptosis in CVDs could provide new therapeutic targets, presenting strong developmental prospects for this field (Fig. 6).

## Conclusions and perspectives

CVDs remain a critical global health challenge, with PCD playing a pivotal role in their progression. CircRNAs have garnered considerable attention for their ability to regulate PCD through various signaling pathways, thereby influencing the development and progression of CVDs. Significant advancements in this area have been achieved. For example, Made et al. constructed a circRNA-miRNA-mRNA dysregulation network in patients with ischemic heart failure, identifying approximately 662 circRNA-miRNA-mRNA interactions in the heart, thus providing novel insights into the pathogenesis of CVDs [190]. Another study in 2023 highlighted the potential of detecting differential circRNA profiles in peripheral circulation via exosomes, underscoring their promise as biomarkers and therapeutic carriers for targeted CVD treatment [191].

Clinical approaches to improving CVD treatment by targeting PCD have already been widely applied. Drugs such as dapagliflozin, carvedilol, dexmedetomidine, simvastatin, nicorandil, and trimetazidine have demonstrated therapeutic effects through various signaling pathways [192–197]. These therapies are effective against conditions like AMI, I/RI and HF, as well as mitigating cardiotoxicity induced by chemotherapeutic agents with strong PCD-inducing capabilities, such as cisplatin and paclitaxel [198–207]. However, the range of available drugs remains limited, highlighting the urgent need to develop new therapeutic strategies for CVD management. Research on circRNAs in CVDs continues to expand, with a primary focus on their role in regulating apoptosis, followed by autophagy and ferroptosis. In contrast, the effects of circRNAs on pyroptosis and necroptosis remain underexplored. While the regulatory roles of circRNAs in various types of cell death during CVD events are significant, and mediated signaling pathways have been partially elucidated, the majority of findings have yet to be translated into clinical applications [208].

Several challenges hinder the clinical translation of circRNA research. First, the lack of a standardized nomenclature for circRNAs complicates communication among researchers [209], as the same circRNA may be referred to by different names in different studies. Second, circRNAs may also play roles in maintaining normal physiological functions, making circRNA-targeted therapies a double-edged sword [21]. Lastly, while the use of circRNAs as diagnostic biomarkers and therapeutic targets represents an emerging direction, their full potential remains to be explored [210].

In summary, the interplay between circRNAs and PCD in the context of CVDs has attracted increasing attention and made substantial progress. This promising field is poised for significant future advancements.

## Data Availability

All data generated or analyzed during this study are included in this article.
